# Supplementary material for: Experimental and Numerical Study of Slug-Flow Velocity Inside Microchannels Through In Situ Optical Monitoring
Source: Micromachines (Basel). 2025 May 17;16(5):586. doi: 10.3390/mi16050586 (PMC12113849; doi:10.3390/mi16050586)
Supplement: Supplementary file 1 [file micromachines-16-00586-s001.zip › micromachines-3602828-Supplementary.pdf]

micromachines

MDPI

Article

Experimental and Numerical Study of Slug-Flow Velocity Inside Microchannels Through In Situ Optical Monitoring

Samuele Moscato [1,2,*], Emanuela Cutuli [1], Massimo Camarda [2,3,4] and Maide Bucolo [1]

1 Department of Electrical Electronic and Computer Science Engineering, University of Catania, Via Santa Sofia 64, 95125 Catania, Italy; emanuela.cutuli@phd.unict.it (E.C.); maide.bucolo@unict.it (M.B.)
2 STLab srl, Via Anapo 53, 95126 Catania, Italy
3 Dipartimento di Fisica e Astronomia 'Ettore Majorana', Università degli Studi di Catania, Via Santa Sofia 64, 95123 Catania, Italy; massimo.camarda@stlab.eu
4 Istituto per la Microelettronica e Microsistemi CNR-IMM, Sezione di Catania, Strada VIII Zona Industriale 5, 95121 Catania, Italy
* Correspondence: samuele.moscato@phd.unict.it

Abstract: Miniaturization and reliable, real-time, non-invasive monitoring are essential for investigating microfluidic processes in Lab-on-a-Chip (LoC) systems. Progress in this field is driven by three complementary approaches: analytical modeling, computational fluid dynamics (CFD) simulations, and experimental validation techniques. In this study, we present an on-chip experimental method for estimating slug flow velocity in microchannels through in-situ optical monitoring. Slug flow involving two immiscible fluids was investigated under both liquid–liquid and gas–liquid conditions via an extensive experimental campaign. The measured velocities were used to determine slug length and key dimensionless parameters, including the Reynolds number and the Capillary number. Comparison with analytical models and CFD simulations revealed significant discrepancies, particularly in gas–liquid flows. These differences are mainly attributed to factors such as gas compressibility, pressure fluctuations, the presence of a liquid film, and leakage flows, all of which substantially affect flow dynamics. Notably, the percentage error in liquid–liquid flows was lower than in gas–liquid flows, largely due to the incompressibility assumption inherent in the model. The high-frequency monitoring capability of the proposed method enables in-situ mapping of evolving multiphase structures, offering valuable insights into slug flow dynamics and transient phenomena that are often difficult to capture using conventional measurement techniques.

Keywords: two-phase flow; microfluidics; micro-optics; computational fluid dynamics; experimental validation.

check for updates

Academic Editor: Célio Fernandes

Received: 7 April 2025
Revised: 12 May 2025
Accepted: 15 May 2025
Published: 17 May 2025

Citation: Moscato, S.; Cutuli, E.; Camarda, M.; Bucolo, M. Experimental and Numerical Study of Slug-Flow Velocity Inside Microchannels Through In Situ Optical Monitoring. *Micromachines* **2025**, *16*, 586. https://doi.org/10.3390/mi16050586

Copyright: © 2025 by the authors. Licensee MDPI, Basel, Switzerland. This article is an open access article distributed under the terms and conditions of the Creative Commons Attribution (CC BY) license (https://creativecommons.org/licenses/by/4.0/).

S1. Strengths, Weaknesses, Opportunities, and Threats (SWOT) Analysis

A *Strengths, Weaknesses, Opportunities, and Threats* (SWOT) analysis was conducted to evaluate and compare the respective advantages of experimental and simulation approaches. As shown in Figure S1, the analysis emphasizes that

- ***Strengths:*** the experimental approach allow for direct observation of the process, offering valuable insights through advanced optical signal measurement techniques that simulations may overlook;

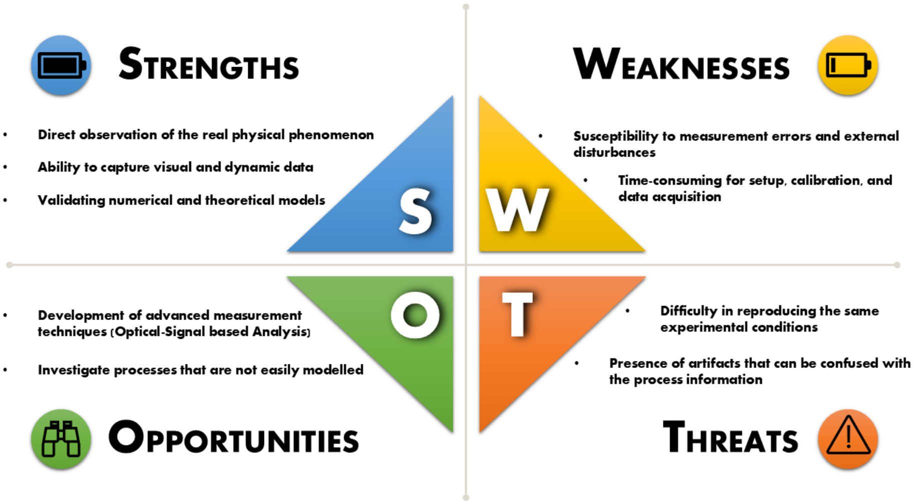

Figure S1. SWOT analysis of the experimental approach compared to simulation: Strengths (S), Weaknesses (W), Opportunities (O), and Threats (T).

- ***Weaknesses:*** experimental procedures require significant time and resources. The complexity of experimental setups can introduce errors and uncertainties;
- ***Opportunities:*** experimental observations offer the opportunity to integrate and validate monitoring technologies, such as the proposed optical signal methodologies, and to investigate complex processes like droplet breakup and interfacial instabilities, which simulations cannot accurately model;
- ***Threats:*** the possible presence of artifacts or anomalies may lead to misinterpretation, and achieving consistent results is challenging due to the inherent variability of multiphase flows.

S2. Micro-Optofluidc Device: Roughness and Resolution

The 3D printed device was manufactured via the Inkjet 3D printing technique, by using the Stratasys Objet260 Connex1 3D printer machine, which is characterized by high accuracy. Thus, in accordance with its TDS (Technical Data Sheet), it is characterized by an XY-accuracy ranging between 100 and 200 μm for the Vero material Photocurable resins. Thus, the 3D printed mold was properly designed for accounting this bias with the aim to achieve the desired channel's width size, i.e., 400 μm. While the Z-resolution, i.e., the employed layer thickness, is equal to 30 μm (Standard quality).
Furthermore, regarding the resulting surface roughness of side walls, it was previously estimated for the considered Inkjet 3D printing machine and Vero material Photocurable resin, by mean of the Atomic Force Microscopy, and it is equal to 10.508 nm in terms of RMS, 6.858 nm in terms of RA and 192.907 nm in terms of Peak to Peak roughness. Thus, being the relative roughness equal to

$$\frac{RA}{Microchannel's\ height} \times 100 = \frac{6.858}{400000} \times 100 \frac{[nm]}{[nm]} = 0.0017\% \quad (1)$$

no instability of the flow inside the channel correlated to the surface roughness for the manufactured device is expected. In fact, a relative roughness < 0.1 % is generally considered hydraulically smooth [1,2].

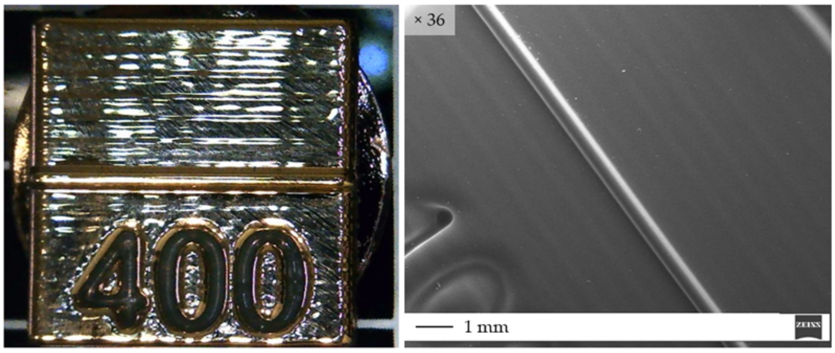

Figure S2. On the left a real picture of the 3D printed specimen produced for calibration featuring a microchannel with a cross-section of 400 μm $\times$ 400 μm, on the right the related acquired SEM micrographs at a magnification of $\times$36.

Finally, Figure S2 shows both a real picture of a 3D-printed calibration sample featuring a microchannel with a cross-section of 400 μm $\times$ 400 μm, produced using the same 3D printing parameters setting, and the corresponding SEM micrograph, which highlights the low surface roughness.

Author Contributions: Conceptualization, S.M., E.C., M.C. and M.B.; methodology, S.M., E.C., M.C. and M.B.; software, S.M.; validation, S.M.; formal analysis, S.M., E.C., M.C. and M.B.; investigation, S.M., E.C., M.C. and M.B.; resources, S.M., E.C., M.C. and M.B.; data curation, S.M.; writing—original draft preparation, S.M., E.C.; writing—review and editing, M.C. and M.B.; visualization, S.M., E.C.; supervision, M.C. and M.B.; project administration, M.B.; funding acquisition, M.B. All authors have read and agreed to the published version of the manuscript.

Funding: This document is the results of the research project funded by the European Union (NextGeneration EU) through the MUR-PNRR project SAMOTHRACE (CUP: E63C220009000022)

Data Availability Statement: Data will be made available on request.

Conflicts of Interest: The authors declare no conflict of interest..

References

1. Zhou, G.; Yao, S.C. Effect of surface roughness on laminar liquid flow in micro-channels. *Applied Thermal Engineering* **2011**, *31*, 228–234.
2. Ranjan, P. Investigations on the flow behaviour in microfluidic device due to surface roughness: a computational fluid dynamics simulation. *Microsystem Technologies* **2019**, *25*, 3779–3789.
